# Sensor Monitoring of Thermal and Vascular Changes During Neoadjuvant Treatment

**DOI:** 10.3390/s26061782

**Published:** 2026-03-12

**Authors:** Catarina C. Zordão, Andrezza M. Flórido, Tamires C. de Almeida, Hélio H. A. Carrara, Andreia Noites, Rinaldo R. J. Guirro, Elaine C. O. Guirro

**Affiliations:** 1 Department of Health Sciences, Faculty of Medicine of Ribeirao Preto, University of São Paulo, Bandeirantes Avenue, 3900, Ribeirao Preto 14049-900, SP, Brazil; andrezzamoraesflorido@alumni.usp.br (A.M.F.); tamires.almeida@usp.br (T.C.d.A.); rguirro@fmrp.usp.br (R.R.J.G.); ecguirro@fmrp.usp.br (E.C.O.G.); 2 Department of Gynecology and Obstetrics, Faculty of Medicine of Ribeirao Preto, University of São Paulo, Ribeirao Preto 14049-900, SP, Brazil; carrara@fmrp.usp.br; 3 Center for Rehabilitation Research (CIR), ESS, Polytechnic of Porto, 4200-072 Porto, Portugal; arn@ess.ipp.pt

**Keywords:** neoadjuvant treatment, thermography, superficial temperature

## Abstract

The physiological impact of neoadjuvant therapy on vascular and thermal responses in breast cancer patients remains poorly understood, despite its clinical relevance for predicting treatment outcomes and managing therapy-related side effects. Sensor-based monitoring technologies, such as thermography and Doppler ultrasound, provide non-invasive approaches to assess circulatory and thermal changes, potentially serving as predictive biomarkers of therapeutic efficacy. This study aimed to evaluate vascular impairment and correlate circulatory alterations with skin surface temperature in women undergoing neoadjuvant therapy for breast cancer. A total of 38 women were enrolled and distributed into two groups: patients receiving eight cycles of neoadjuvant chemotherapy and healthy controls. Thermographic imaging was employed to measure upper-limb surface temperature, while Doppler ultrasound assessed arterial and venous blood flow in the cubital fossa. Paired Student’s *t*-tests compared experimental moments (C1, C5, C8), with normality assessed from difference scores (Δ) and results expressed as mean differences with 95% CIs (*p* < 0.05, two-tailed). Associations between surface temperature and arterial blood flow were examined using simple linear regression (R^2^, F-statistic, β, *p*-values). Analyses were performed in SPSS 20.0 (SPSS Inc., Chicago, IL, USA). Significant increases in surface temperature (*p* < 0.001) and blood flow velocity (*p* < 0.004) were observed in patients compared with controls prior to therapy, suggesting pre-existing vascular and thermal dysregulation. Neoadjuvant therapy significantly altered thermal and vascular dynamics, reinforcing the utility of sensor-based monitoring to capture subtle physiological responses during treatment.

## 1. Introduction

Neoadjuvant treatment of breast cancer, a disease with high incidence and prevalence worldwide, is considered an important management approach for localized disease in order to understand its biology, in addition to investigating new treatment strategies [1]. Neoadjuvant chemotherapy, designed to be used before surgical intervention for tumor resection, has received significant attention in research and clinical practice. The procedure has advantages, such as increasing the overall survival rate for patients with a good response to therapy, in addition to allowing more conservative surgeries due to the reduction in tumor volume, with a consequent decrease in morbidity and mortality [2].

Despite being a frequently used therapy and the advantages of neoadjuvant treatment, chemotherapy triggers important side effects in the body, due to peripheral vascular and neurotoxic effects, loss of muscle mass, and fatigue [3,4]. It can also interfere with posture, balance and function [5], in addition to affecting changes in arterial and venous blood flow in the affected limb [6], increasing circulatory complications in patients who already have an increased risk of developing heart and vascular diseases [7].

Infrared thermography, a non-invasive and non-radioactive sensor-based analysis, is considered a reliable complementary resource in the detection of breast cancer [8] and is also used as a tool to monitor the response rate to chemotherapy [9]. It has high sensitivity for detecting temperature changes on the body surface, making it an important field of study for monitoring cancer treatment [10]. On the other hand, Doppler ultrasound, another sensor technology, evaluates macrocirculation and is often used to assess blood flow in large vessels, such as arteries and veins. Both sensor-based methods have specific applications and can complement each other in circulatory assessment [11].

The objective of the study was to evaluate blood circulation impairment resulting from neoadjuvant therapy in women affected by breast cancer, as well as to correlate circulatory changes with skin surface temperature. It aims to increase knowledge regarding vascular dysfunctions and to improve forms of therapeutic intervention in the face of adaptive changes and their consequences during and after breast cancer treatment.

## 2. Materials and Methods

### 2.1. Study Design

The project was approved by the Research Ethics Committee of the Hospital das Clínicas of the Faculty of Medicine of Ribeirão Preto of the University of São Paulo, CEP-HCFMRP-USP, through process 4.188.751/2020 and with the Helsinki Declaration of 1975, as revised in 2000. All patients consented and signed the Terms of Free and Informed Consent.

The sample was calculated based on the study by Rezende et al. (2017) [12], considering the mean measured arterial flow velocity as the outcome variable, with a statistical power of 80% and an alpha error of 0.05. The program used was G*Power 3 (Christian-Albrechts-Universität, Kiel, Germany), resulting in *n* = 19 per group.

### 2.2. Patient Recruitment

The sample of this study was composed of 38 women with a mean age of 44 (±12.9) years, divided into two groups: the control group (CG), composed of women without a diagnosis of breast cancer and any previous treatment for any neoplasm; and a group of women undergoing neoadjuvant therapy (NG), composed of women indicated for neoadjuvant chemotherapy as a treatment for breast cancer.

Neoadjuvant therapy was carried out in total through eight cycles of chemotherapy, with an interval of 15 to 21 days between each cycle, with the infusion of anthracycline medication in the initial four cycles, followed by taxane drugs in the final four cycles. The evaluations of the neoadjuvant therapy group were carried out at three different times: before the first cycle of chemotherapy (C1), before the fifth cycle (C5) and after the eighth cycle (C8) of neoadjuvant chemotherapy. In the control group, the evaluation was performed only once.

Women over the age of 21, diagnosed with breast cancer, treated for unilateral breast cancer, and without the disease (for the control group), in good physical and mental health were included. Patients with associated diseases that could interfere with the evaluation were not included, such as skin disorders, ongoing radiotherapy treatment, bilateral axillary dissection, diagnosis of metastasis, functional changes in the upper limbs before breast cancer treatment, hemodynamically significant disease, vascular changes, uncontrolled arterial hypertension, uncontrolled diabetes mellitus, cognitive changes, and complications with venous access for medication infusion; those who did not agree to sign the free and informed consent form or who did not meet the inclusion criteria described were also excluded.

The clinical assessment used a standard anamnesis consisting of the sociodemographic profile and anthropometric data. The assessments were performed by the same evaluator and repeated consistently according to a standardized procedure at all evaluation time points. Each evaluation began with the measurement of skin temperature, due to the need for room acclimatization, followed by the assessment of blood flow velocity. All examinations were conducted in the morning to minimize the effects of hormonal variations during the day [13].

### 2.3. Data Collection

#### 2.3.1. Infrared Thermography

The evaluation of superficial blood circulation was carried out using the infrared temperature of the upper limbs using an FLIR^®^ thermograph, model T300 W/25۫ (FLIR^®^ Systems, Wilsonville, OR, USA), with a sensitivity of 0.1 °C, plane matrix focal point (FPA) of 320/240 points, emissivity of 0.98, and focal length of 2 m, and stabilized for 10 min before the exams, with an intraclass correlation coefficient (ICC) 0.88 [14].

The patients underwent 15 min of adaptation to the room temperature without the incidence of air and direct sunlight. They were also advised not to approach electrical equipment that generates heat, to avoid heat sources within the collection environment, and to light the environment using fluorescent lamps. They were also instructed to avoid smoking; taking hot baths or showers; using topical agents, creams, and powders; practicing vigorous exercise; and ingesting stimulants, such as caffeine, chocolates or nasal decongestants, for two hours before the day of collections [15].

**Figure 1:**
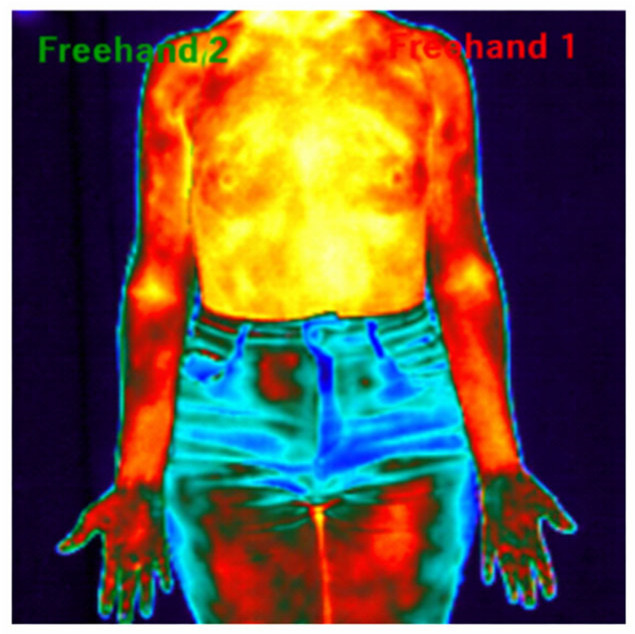
Infrared thermography: Thermographic analysis using the ROI of a patient with a tumor in the left breast before the start of neoadjuvant treatment.

The images were recorded standing up, with weight distributed on both lower limbs, the upper limbs relaxed along the body, and the anterior region of the limb facing forward (Figure 1). The removal of clothing, decorations, and accessories was advised to avoid interference with the results, according to criteria observed in a study of the related population [8].

#### 2.3.2. Assessment of Blood Circulation

**Figure 2:**
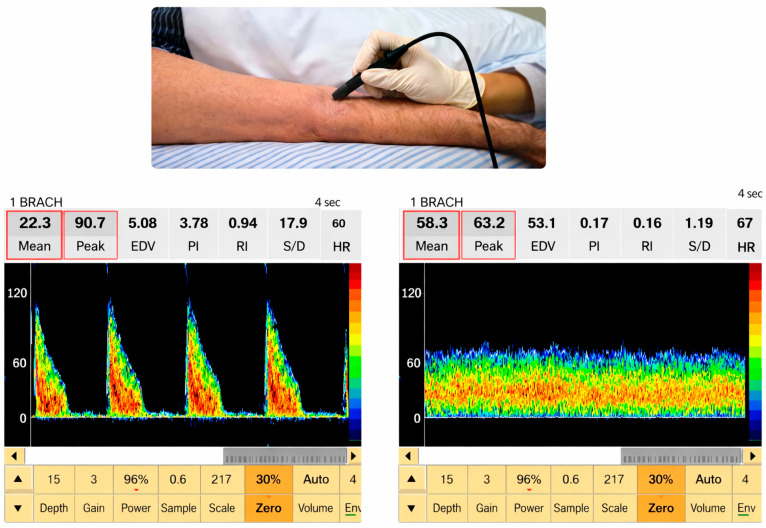
Image illustrating the positioning of the probe during the assessment procedure, as well as the visualization of the image and blood flow parameters obtained during the evaluation of the cubital artery and vein of the upper limb.

The brachial arterial and venous circulation assessment was carried out using SONARA/Tek Doppler ultrasound (Nicolet Vascular, Madison, WI, USA), with a 4 MHz probe, with ICC 0.72 [16]. The arterial and venous blood flow velocity was analyzed with the transducer positioned at 45 °C in relation to the evaluated blood vessel, with the skin smeared with water-soluble gel. Confirmation of the appropriate location for evaluation was detected through observation of the spectral image and sound signals produced by the equipment (Figure 2) [17].

The blood flow velocity of the involved limb was evaluated in the supine position with the probe positioned in the cubital fossa in a controlled environment at 23 °C after resting for five minutes in the supine position [16].

Arterial impairment was assessed using the ankle–brachial index (ABI), carried out with an automatic oscillometric sphygmomanometer from the brand G-TECH^®^ (G-TECH^®^, Accumed, Duque de Caxias, RJ, Brazil), with measurements obtained in the supine position after five minutes of rest, considering the ratio between the highest systolic pressures of the posterior tibial and brachial arteries bilaterally [18]. Values between 0.9 and 1.4 were considered normal for ABI, with indexes greater than 1.4 representing increased artery resistance and those less than or equal to 0.9 demonstrating the presence of peripheral arterial disease [19].

#### 2.3.3. Member Dominance Assessment

Limb preference was determined through the Edinburgh Handedness Inventory, characterized by responses to a questionnaire relating to lateral preference in 12 daily activities [20].

In patients, limb classification was based on tumor laterality (ipsilateral/contralateral), which does not necessarily correspond to limb dominance; in controls, classification was based on dominance (dominant/non-dominant). This distinction was necessary because tumor laterality does not consistently overlap with limb dominance.

### 2.4. Statistical Analysis

Within-group comparisons across experimental moments (C1, C5, and C8) were conducted using paired Student’s *t*-tests. The assumption of normality was assessed based on the distribution of the difference scores (Δ). Results are reported as mean differences (Δ) with corresponding 95% confidence intervals (95% CI). All tests were two-tailed, and statistical significance was set at *p* < 0.05.

Simple linear regression analyses were performed to evaluate the association between surface temperature and arterial blood flow in the upper limbs at each experimental moment. The coefficient of determination (R^2^), F-statistic, regression coefficients (β), and corresponding *p*-values were calculated. Statistical significance was established at *p* < 0.05.

All analyses were conducted using SPSS software (version 20.0, SPSS Inc., Chicago, IL, USA).

## 3. Results

Thirty-eight women were evaluated, all meeting the inclusion criteria and undergoing all necessary assessments. Nineteen volunteers were included in the study in the group of women diagnosed with breast cancer undergoing neoadjuvant therapy (NG), who completely completed the chemotherapy cycles, with the medication infusion occurring in veins located in the hand of the upper limb ipsilateral to the breast neoplasia, with only two cases in which the last four cycles of chemotherapy were infused through a catheter fully implanted in the vena cava (portocath), and 19 volunteers were included in the control group (CG). The sample characterization data are presented in Table 1, and the ABI was classified as normal for both groups (>0.9 and <1.3).

The average surface temperature data for both upper limbs are described in Table 2, with upper limb one (UL1—*n* = 19) being related to the limb ipsilateral to the breast neoplasm and upper limb two (UL2—*n* = 19) being related to the limb contralateral to the breast cancer. Breast neoplasia in NG. For the CG, UL1 and UL2 are linked to member preference, respectively, with the highest prevalence being the right preferred member. Significant differences (*p* < 0.05) were found for both upper limbs, comparing the CG with the NG for the evaluations before C1, referring to the evaluation before chemotherapy treatment, and after C8, referring to the review after the last cycle of chemotherapy. There was no significant difference between the evaluation steps of neoadjuvant therapy.

Significant differences (*p* < 0.05) were found between CG and NGC1 for both upper limbs and between CG and NGC5 only in the upper limb ipsilateral to the neoplasia regarding the mean arterial flow velocity of the upper limbs (Table 3). There were no significant differences when comparing the evaluation steps neoadjuvant chemotherapy with the velocity of arterial blood flow in the brachial artery.

A significant difference (*p* < 0.05) regarding the average venous flow velocity of the upper limbs was observed between the CG and the NGC5 only for the upper limb ipsilateral to the neoplasm, with no significant differences occurring between the groups at other times. There were no significant differences when comparing the times of receiving neoadjuvant chemotherapy in the venous blood flow velocity of the brachial vein (Table 4).

No significant difference was found between the values in the CG and NG groups at all times evaluated when comparing temperature and blood flow velocity between both the preferred and non-preferred sides and between the sides ipsilateral and contralateral to the neoplasm (Table 5).

The correlation between surface temperature and arterial blood flow velocity in the upper limbs was assessed using linear regression, and no significant correlation was observed between the samples. Although a trend toward a positive association was observed at C5, it did not reach statistical significance (Table 6).

## 4. Discussion

The study in question investigated the impact of neoadjuvant chemotherapy on the temperature and speed of blood flow in the upper limbs of women with breast cancer, evaluated at three moments: before the first cycle (C1), in the fifth cycle (C5) and after completion of cycles (C8).

The significant findings for both upper limbs compared both groups (subjected to neoadjuvant therapy—NG—and control—CG) for the evaluations before the first cycle of neoadjuvant chemotherapy (pre-C1) and after the eighth after C8, referring to evaluation after the last cycle of chemotherapy. There was no significant difference between the evaluation stages of neoadjuvant treatment. The difference between groups may be related to the fact that infiltrative carcinoma promotes hypervascularization, hypermetabolism and neo-angiogenesis, causing an increase in local temperature [21]. Abnormal vessel function is characteristic of cancer and inflammatory diseases [22], and is associated with the regulation of cellular mediators responsible for the pathological signaling of endothelial cells, increasing the circulatory process [23], and the consequent increase in adjacent tissue temperature, caused by inflammatory cytokines [24].

In addition to the increase in temperature at the tumor site, observed in studies [9,10], a reduction in temperature was also observed throughout neoadjuvant treatment. Infrared imaging captures physiological changes related to locations with increased temperature, associated with vasodilation, hypermetabolism, hyperperfusion and hypervascularization, which generate a heat source [10]. Thermography can be used to monitor the response to neoadjuvant therapies such as chemotherapy and has been widely disseminated in the literature [10,25], a fact that corroborates our findings. Fredslund et al., 2021 [26], observed early vasodilation related to chemotherapy treatment.

Significant differences in arterial flow velocity were observed for both upper limbs between the groups (NG and CG) for the pre-C1 and post-C8 moments. As in the temperature data, no significant difference was found between the evaluation stages of neoadjuvant treatment. Vascular toxicity is currently considered a frequent adverse effect of current anticancer chemotherapies resulting from endothelial dysfunction [27] and may be the explanation for our findings.

The increase in blood flow velocity was found in the upper limbs of patients undergoing neoadjuvant treatment for breast cancer compared to those not undergoing treatment. Blood circulation through the vascular system represents the main route of infusion and transport of drugs, especially chemotherapy drugs, causing damage to the circulatory system [28]. Part of vascular growth is mediated by angiogenesis, which is involved in endothelial cell proliferation, migration and maturation [29]. Endothelial cells significantly decrease their ability to form vascular structures and increase their sensitivity to chemotherapy-related antiangiogenic and vascular disruption effects, which harm the vascular endothelium [30].

Meta-analysis [31] detected that arterial stiffness in cancer patients increased significantly after antineoplastic therapy, demonstrating the significant relationship between the finding and treatment with antineoplastic therapy, with several clinical implications. These observations expand our understanding of the effects of antineoplastic therapy on the cardiovascular system beyond the heart, demonstrating that increases in arterial stiffness are detectable soon after treatment and can persist for years.

The conclusions of this study are limited to the analyses performed, which focused on skin temperature and blood flow velocity as indicators of the physiological impact of neoadjuvant therapy. Vessel morphology was not included, which highlights an opportunity for future research to explore whether morphological parameters add further relevance or if functional sensor-based measurements alone are sufficient for clinical monitoring. Although the sample size was determined by a priori calculation and is adequate for the proposed analyses, future studies with larger cohorts and expanded analytical approaches may further substantiate and deepen the present findings.

## 5. Conclusions

This study demonstrated a complementary relationship between microcirculatory findings, assessed by infrared thermography, and macrocirculatory parameters, represented by arterial and venous flow velocity analysis using Doppler ultrasound. The combined sensor-based approaches consistently revealed vascular and thermal alterations associated with neoadjuvant therapy. Based on the parameters assessed, it can be concluded that neoadjuvant therapy influences both skin temperature and blood circulation velocity. These results underscore the potential of integrating multimodal sensor technologies into oncological monitoring, providing valuable insights into treatment-related physiological characterization and supporting predictive evaluation of therapeutic outcomes.

## Figures and Tables

**Table 1:** Sample characterization.

Variable	Average/Standard Deviation/Percentage		
	NG	CG	*p*
Number of participants	19	19	-
Age (years)	44.75 (±10.72)	43 (±9.54)	0.004 *
BMI (kg/cm^2^)	25.78 (±2.97)	24.42 (±3.98)	0.004 *
Affected breast (R/L) (%)	35/65	-	-
Preferred upper limb (R/L) (%)	85/15	95/5	-
Family history of cancer (%)	60	75	-

* Indicates statistically significant differences (*p* < 0.05).

**Table 2:** Mean and median temperature of the upper limbs of women with and without neoadjuvant chemotherapy.

Variable	Group	Mean	Median	SD	Min	Max	Δ + 95% CI	*p*
Temp UL1 (°C)	CG	29.82	29.73	1.09	28.37	32.27	2.365 [1.62; 3.10]	0.001 *
	NGC1	32.15	32.48	1.21	28.87	33.83		
Temp UL2 (°C)	CG	29.21	29.35	2.23	21.43	32.23	−2.940 [−4.03; −1.85]	0.001 *
	NGC1	32.15	32.27	1.16	29.43	33.67		
Temp UL1 (°C)	CG	29.82	29.73	1.09	28.37	32.27	1.171 [0.38; 1.96]	0.121
	NGC5	31.13	31.25	1.41	28.47	33.40		
Temp UL2 (°C)	CG	29.21	29.35	2.23	21.43	32.23	−1.795 [−2.86; −0.72]	0.071
	NGC5	31.16	31.45	1.36	28.47	33.23		
Temp UL1 (°C)	CG	29.82	29.73	1.09	28.37	32.27	1.647 [0.89; 2.40]	0.001 *
	NGC8	31.47	31.58	0.9	29.27	32.73		
Temp UL2 (°C)	CG	29.21	29.35	2.23	21.43	32.23	−2.339 [−3.44; −1.23]	0.002 *
	NGC8	31.55	31.88	1.01	29.73	32.93		
Temp UL1 (°C)	NGC1	32.15	32.48	1.21	28.87	33.83	1.191 [0.35; 2.02]	0.008
	NGC5	31.13	31.25	1.41	28.47	33.40		
Temp UL2 (°C)	NGC1	32.15	32.27	1.16	29.43	33.67	1.153 [0.35; 1.94]	0.007
	NGC5	31.16	31.45	1.36	28.47	33.23		
Temp UL1 (°C)	NGC1	32.15	32.48	1.21	28.87	33.83	0.680 [0.12; 1.23]	0.018
	NGC8	31.47	31.58	0.9	29.27	32.73		
Temp UL2 (°C)	NGC1	32.15	32.27	1.16	29.43	33.67	0.600 [0.12; 1.07]	0.016
	NGC8	31.55	31.88	1.01	29.73	32.93		
Temp UL1 (°C)	NGC5	31.13	31.25	1.41	28.47	33.40	−0.337 [−1.00; 0.33]	0.302
	NGC8	31.47	31.58	0.9	29.27	32.73		
Temp UL2 (°C)	NGC5	31.16	31.45	1.36	28.47	33.23	−0.392 [−1.02; 0.23]	0.206
	NGC8	31.55	31.88	1.01	29.73	32.93		

SD: Standard deviation; UL1: upper limb ipsilateral to the neoplasm in the neoadjuvant group and dominant in the control group; UL2: upper limb contralateral to the neoplasm in the neoadjuvant group and non-dominant in the control group; CG: control group; NGC1: neoadjuvant therapy group for pre-1st-cycle breast cancer; NGC5: neoadjuvant therapy group for pre-5th-cycle breast cancer; NGC8: neoadjuvant therapy group for breast cancer after the 8th cycle; *p*: *p*-value. * Indicates statistically significant differences (*p* < 0.05).

**Table 3:** Mean and median arterial flow velocity in the upper limbs of women with and without neoadjuvant chemotherapy.

Variable	Group	Mean	Median	SD	Min	Max	Δ + 95% CI	*p*
Flow vel UL1 (cm/s)	CG	9.87	8.98	4.12	5.54	24.27	4.149 [0.26; 8.03]	0.038 *
	NGC1	14.02	11.63	6.31	8.53	30.13		
Flow vel UL2 (cm/s)	CG	10.37	9.60	2.68	6.90	15.37	−3.853 [−6.32; −1.38]	0.004 *
	NGC1	14.22	14.36	5.07	7.71	28.13		
Flow vel UL1 (cm/s)	CG	9.87	8.98	4.12	5.54	24.27	3.652 [0.26; 7.03]	0.036 *
	NGC5	13.52	11.41	6.11	7.44	31.43		
Flow vel UL2 (cm/s)	CG	10.37	9.60	2.68	6.90	15.37	−2.431 [−5.00; 0.14]	0.064
	NGC5	12.80	12	4.71	6.26	24.10		
Flow vel UL1 (cm/s)	CG	9.87	8.98	4.12	5.54	24.27	2.861 [−0.83; 6.56]	0.085
	NGC8	12.78	11.66	4.78	6.53	21.77		
Flow vel UL2 (cm/s)	CG	10.37	9.60	2.68	6.90	15.37	−1.186 [−4.03; 1.66]	0.392
	NGC8	11.55	10.01	4.73	3.99	18.88		
Flow vel UL1 (cm/s)	NGC1	14.02	11.63	6.31	8.53	30.13	0.497 [−4.03; 5.02]	0.936
	NGC5	13.52	11.41	6.11	7.44	31.43		
Flow vel UL2 (cm/s)	NGC1	14.22	14.36	5.07	7.71	28.13	1.422 [−1.44; 4.29]	0.516
	NGC5	12.80	12	4.71	6.26	24.10		
Flow vel UL1 (cm/s)	NGC1	14.02	11.63	6.31	8.53	30.13	0.343 [−2.988; 3.67]	0.711
	NGC8	12.78	11.66	4.78	6.53	21.77		
Flow vel UL2 (cm/s)	NGC1	14.22	14.36	5.07	7.71	28.13	2.598 [−0.82 to 6.02]	0.267
	NGC8	11.55	10.01	4.73	3.99	18.88		
Flow vel UL1 (cm/s)	NGC5	13.52	11.41	6.11	7.44	31.43	1.028 [−1.79; 3.85]	0.711
	NGC8	12.78	11.66	4.78	6.53	21.77		
Flow vel UL2 (cm/s)	NGC5	12.80	12	4.71	6.26	24.10	−1.335 [−3.04; 0.37]	0.122
	NGC8	11.55	10.01	4.73	3.99	18.88		

SD: Standard deviation; flow vel: average speed of blood flow in the vessel; UL1 (*n* = 19) is the upper limb ipsilateral to the neoplasm in the neoadjuvant group (which can be dominant or non-dominant) and the dominant limb in the control group; UL2 (*n* = 19) is the upper limb contralateral to the neoplasm in the neoadjuvant group (which can also be dominant or non-dominant) and the non-dominant limb in the control group; CG: control group; NGC1: neoadjuvant therapy group for pre-1st-cycle breast cancer; NGC5: neoadjuvant therapy group for pre-5th-cycle breast cancer; NGC8: neoadjuvant therapy group for breast cancer after the 8th cycle; *p*: *p*-value. * Indicates statistically significant differences (*p* < 0.05).

**Table 4:** Mean and median venous flow velocity in the upper limbs of women with and without neoadjuvant chemotherapy.

Variable	Group	Mean	Median	SD	Min	Max	Δ + 95% CI	*p*
Flow vel UL1 (cm/s)	CG	8.90	7.53	5.36	2.27	22.40	2.844 [−1. 93; 5.88]	0.053
	NGC1	11.75	10.06	4.78	6.22	25.87		
Flow vel UL2 (cm/s)	CG	10.16	10.15	4.38	3.72	22.86	−3.539 [−7.52; 0.44]	0.059
	NGC1	13.70	11.33	7.05	3.36	27.30		
Flow vel UL1 (cm/s)	CG	8.90	7.53	5.36	2.27	22.40	3.497 [0.25; 6.74]	0.033 *
	NGC5	12.40	11.26	6.62	3.99	27.80		
Flow vel UL2 (cm/s)	CG	10.16	10.15	4.38	3.72	22.86	−1.639 [−4.89; 1.61]	0.546
	NGC5	11.80	9.78	6.94	3.90	29.60		
Flow vel UL1 (cm/s)	CG	8.90	7.53	5.36	2.27	22.40	0.219 [−3.38; 3.82]	0.845
	NGC8	9.44	8.80	3.97	3.63	19.03		
Flow vel UL2 (cm/s)	CG	10.16	10.15	4.38	3.72	22.86	3.488 [−0.67; 7.64]	0.071
	NGC8	13.90	12.15	7.45	4.81	30.10		
Flow vel UL1 (cm/s)	NGC1	11.75	10.06	4.78	6.22	25.87	−0.652 [−3.99; 2.69]	0.809
	NGC5	12.40	11.26	6.62	3.99	27.80		
Flow vel UL2 (cm/s)	NGC1	13.70	11.33	7.05	3.36	27.30	1.900 [−2.54; 6.34]	0.334
	NGC5	11.80	9.78	6.94	3.90	29.60		
Flow vel UL1 (cm/s)	NGC1	11.75	10.06	4.78	6.22	25.87	2.460 [−0.639; 5.56]	0.145
	NGC8	9.44	8.80	3.97	3.63	19.03		
Flow vel UL2 (cm/s)	NGC1	13.70	11.33	7.05	3.36	27.30	−0.071 [−4.49; 4.354	0.948
	NGC8	13.90	12.15	7.45	4.81	30.10		
Flow vel UL1 (cm/s)	NGC5	12.40	11.26	6.62	3.99	27.80	3.199 [−0.55; 6.95]	0.085
	NGC8	9.44	8.80	3.97	3.63	19.03		
Flow vel UL2 (cm/s)	NGC5	11.80	9.78	6.94	3.90	29.60	−1.702 [−6.98; 3.58]	0.420
	NGC8	13.90	12.15	7.45	4.81	30.10		

SD denotes standard deviation; flow vel represents the average speed of blood flow in the vessel; UL1 (*n* = 19) is the upper limb ipsilateral to the neoplasm in the neoadjuvant group (which can be dominant or non-dominant) and the dominant limb in the control group; UL2 (*n* = 19) is the upper limb contralateral to the neoplasm in the neoadjuvant group (which can also be dominant or non-dominant) and the non-dominant limb in the control group; CG refers to the control group; NGC1 is the neoadjuvant therapy group for pre-1st-cycle breast cancer; NGC5 is the neoadjuvant therapy group for pre-5th-cycle breast cancer; NGC8 is the neoadjuvant therapy group for breast cancer after the 8th cycle; *p* indicates the *p*-value. * Indicates statistically significant differences (*p* < 0.05).

**Table 5:** Comparison between temperature, arterial and venous flow, and dominant and non-dominant limbs ipsilateral to the breast tumor and contralateral.

		NG UL1 e NG UL2 (Pre-C1)	NG UL1 e NG UL2 (Pre-C5)	NG UL1 e NG UL2 (Post-C8)	CG UL1 e CG UL2
Temperature	z	−0.492	−1.244	−1.356	−1.547
	*p*	0.623	0.214	0.175	0.122
Arterial flow	z	−0.684	−0.644	−1.502	−0.644
	*p*	0.494	0.520	0.133	0.520
Venous flow	z	−0.724	−0.201	−0.265	−1.046
	*p*	0.469	0.841	0.008	0.295

NG: Neoadjuvant therapy group for breast cancer; CG: control group; UL1 is the upper limb ipsilateral to the neoplasm in the neoadjuvant group (which can be dominant or non-dominant) and the dominant limb in the control group; UL2 is the upper limb contralateral to the neoplasm in the neoadjuvant group (which can also be dominant or non-dominant) and the non-dominant limb in the control group; z: z-score; *p*: *p*-value.

**Table 6:** Correlation between the surface temperature variables and the average blood flow velocity of the upper limbs between the different times evaluated in the neoadjuvant and control groups.

		NGC1	NGC5	NGC8	CG
Temperature x arterial blood flow velocity	β	0.18	0.41	0.07	1.37
	F	0.54	3.64	0.08	2.46
	R^2^	0.032	0.168	0.005	0.127
	*p*	0.472	0.074	0.780	0.135
	95% CI β	[−0.32; 0.68]	[−0.04; 0.86]	[−0.47; 0.61]	[−0.47; 3.22]

NGC1: Neoadjuvant therapy group for pre-1st-cycle breast cancer; NGC5: neoadjuvant therapy group for pre-5th-cycle breast cancer; NGC8: neoadjuvant therapy group for breast cancer after the 8th cycle; group; CG: control group; β: β-value; F: f-value; R^2^: coefficient of determination; *p*: *p*-value.

## Data Availability

The datasets presented in this article are not readily available because the participants of this study did not give written consent for their data to be shared publicly due to the sensitive nature of the research. Requests to access the datasets should be directed to the corresponding author.

## Abbreviations

The following abbreviations are used in this manuscript:

ABI	Ankle–brachial index
BMI	Body mass index
C1	First cycle of chemotherapy
C5	Fifth cycle of chemotherapy
C8	Eighth cycle of chemotherapy
CG	Control group
ICC	Intraclass correlation coefficient
GLPI	Global Lateral Preference Inventory
NG	Group of women undergoing neoadjuvant treatment for breast cancer
UL1	The limb ipsilateral to the breast neoplasm in the group of women undergoing neoadjuvant treatment for breast cancer and the dominant upper limb in the control group
UL2	the limb contralateral to the breast cancer group of women undergoing neoadjuvant treatment for breast cancer and the non-dominant upper limb in the control group
